# Expression of Manganese Transporters ZIP8, ZIP14, and ZnT10 in Brain Barrier Tissues

**DOI:** 10.3390/ijms251910342

**Published:** 2024-09-26

**Authors:** Shannon Morgan McCabe, Ningning Zhao

**Affiliations:** School of Nutritional Sciences and Wellness, The University of Arizona, Tucson, AZ 85721, USA; morgans3@arizona.edu

**Keywords:** manganese, ZIP8, ZIP14, ZnT10, blood–cerebrospinal fluid barrier, blood–brain barrier

## Abstract

Manganese (Mn) is an essential trace mineral for brain function, but excessive accumulation can cause irreversible nervous system damage, highlighting the need for proper Mn balance. ZIP14, ZnT10, and ZIP8 are key transporters involved in maintaining Mn homeostasis, particularly in the absorption and excretion of Mn in the intestine and liver. However, their roles in the brain are less understood. The blood–cerebrospinal fluid barrier and the blood–brain barrier, formed by the choroid plexus and brain blood vessels, respectively, are critical for brain protection and brain metal homeostasis. This study identified ZIP14 on the choroid plexus epithelium, and ZIP8 and ZnT10 in brain microvascular tissue. We show that despite significant Mn accumulation in the CSF of *Znt10* knockout mice, ZIP14 expression levels in the blood–cerebrospinal fluid barrier remain unchanged, indicating that ZIP14 does not have a compensatory mechanism for regulating Mn uptake in the brain in vivo. Additionally, Mn still enters the CSF without ZIP14 when systemic levels rise. This indicates that alternative transport mechanisms or compensatory pathways ensure Mn balance in the CSF, shedding light on potential strategies for managing Mn-related disorders.

## 1. Introduction

Manganese (Mn) is an essential trace metal that causes neurotoxicity when its concentration rises in the brain. Mn accumulates in dopaminergic motor centers such as the striatum and globus pallidus [1,2] and other brain areas responsible for movement, such as the cerebellum and hippocampus [3]. Long-term Mn exposure causes extrapyramidal motor effects resembling Parkinson’s disease symptoms [4]. The exact mechanism of metal accumulation in the brain remains unknown, but recent studies suggest that Mn-specific transporters within two brain barriers are involved in central nervous system Mn homeostasis [5,6,7,8]. Within the regulated environment of the brain barriers, cerebrospinal fluid (CSF), which fills the brain ventricles, is continuous with the interstitial fluid, which fills small gaps between cells in the brain parenchyma [9]. Together, the movements of CSF and interstitial fluid provide the ideal environment for normal brain function by delivering electrolytes and nutrients to brain cells and removing metabolites and waste products from the brain tissue. The blood–brain barrier and the blood–CSF barrier separate the brain, CSF, and interstitial fluid from the rest of the body. Microvascular endothelial cells within the brain regulate the passage of materials from the blood vessel to the interstitial fluid via tight junctions and highly selective transporters. This endothelium makes up the blood–brain barrier. In the CSF-filled ventricles, the choroid plexus consists of a folded sheet, or bundles, of specialized cuboidal epithelial cells all connected to a branching structure of capillaries traveling through the four brain ventricles [10]. The blood vessels are fenestrated, but the endothelium is surrounded by a layer of epithelial cells connected to each other by tight junctions. The epithelial layer of this tissue serves as the blood–CSF barrier.

The brain barriers utilize transport proteins to regulate the influx of essential materials from circulating blood that are required for brain function. The polarized endothelial layer of the blood–brain barrier and the choroid plexus epithelium of the blood–CSF barrier, with their luminal (blood-facing) and abluminal (brain-facing) membranes, provide two restrictive barriers to control the movement of nutrients and metabolites into and out of the brain.

The mechanism of brain Mn homeostasis involving both brain barriers is currently unknown. Several studies have provided insight into Mn transport and distribution in vivo. In a study of Mn distribution after intraperitoneal Mn injection in adult rats, the choroid plexus accumulated 5–10 times more Mn per gram of tissue than Mn per mL of circulating blood. Additionally, the CSF and cortex Mn levels remained lower than blood levels at all doses tested, implicating the choroid plexus as a protective barrier, which quickly sequesters Mn to protect the brain and CSF from exposure [11]. Additionally, Mn-enhanced MRI studies in animals have shown that Mn can be observed quickly accumulating in the choroid plexus. In a study on rats, within 5 min of infusing with MnCl_2_, the choroid plexus quickly became the site of Mn accumulation, with gradual spreading of the Mn-associated signal to the CSF and ventricle-adjacent brain areas [12]. In a human study with healthy subjects, a Mn-based contrast agent (Mangafodipir) was administered intravenously, resulting in the significant accumulation of Mn signals in under an hour [13]. Taken together, these results emphasize an important role for the blood–CSF barrier in brain Mn homeostasis.

While studies in vivo exemplify the likelihood of Mn homeostasis occurring primarily at the choroid plexus, cell culture experiments provide further evidence that the epithelial and endothelial cells of the blood–CSF barrier and the blood–brain barrier express specific transporters to regulate Mn uptake into the cell. Metal transport proteins ZIP8, ZIP14, and ZnT10 are expressed throughout the body and provide homeostatic control of Mn levels from the liver and intestine (reviewed in [14]). Encoded by genes *SLC39A8* and *SLC39A14*, respectively, ZIP8 and ZIP14 are importers responsible for the movement of metals from extracellular space into the cytosol, while ZnT10, encoded by gene *SLC30A10*, is expressed on the plasma membrane as a metal exporter, excreting cytosolic metals into the extracellular environment. Originally thought to be promiscuous divalent metal importers, ZIP8 and ZIP14 may preferentially transport Mn over other divalent metals. A competition metal transport assay for ZIP8 reveals a higher affinity for Mn than cadmium, a toxic non-essential metal, suggesting Mn is the primary biological substrate for ZIP8 [15]. Results from gene knockout (KO) mouse studies of these metal transporters also show the specificity of ZIP8 and ZIP14 for Mn transport. In a study using *Zip14* KO mice, brain tissue drastically accumulated Mn, with only minimal changes in Zn and no change in Fe levels [16]. Due to their roles in absorbing and excreting Mn via the liver and intestine, ZIP8 and ZIP14 have recently been studied for their potential roles in brain barrier Mn transport. A recent study describes ZIP8 and Mn transport at the blood–brain barrier, in which brain Mn levels were markedly reduced in mice with ZIP8 knocked out in brain blood vessels [8]. This finding suggests that ZIP8 at the blood–brain barrier is necessary for normal brain Mn levels; however, the protein expression of ZIP8 in the choroid plexus was not quantified, so Mn transport was not investigated at the blood–CSF barrier.

In a study of blood–brain barrier Mn transport, human brain microvascular endothelial cells were grown as a polarized monolayer with tight junctions using the Transwell system. Endothelial cells were found to express ZIP8 and ZIP14 on both the apical and basolateral sides, and Mn uptake was correlated with the relative level of ZIP8 and ZIP14 expression [5]. This is consistent with an earlier study that established Mn transport across the endothelium as an active transport process [17]. Two recent studies of Mn transport across the choroid plexus epithelium establish that Mn flux is primarily an active process, and ZIP14 is involved in Mn uptake. First, a model of choroid plexus epithelium using polarized primary choroid plexus epithelial cells on Transwells reveals that Mn transport is an active process from the basolateral side, or in the blood-to-CSF direction [18]. HIBCPP cells, an immortalized cell line of choroid plexus epithelial cells commonly used in choroid plexus permeability studies, were cultured on Transwells to form a continuous monolayer. The cell surface protein biotinylation assay revealed that ZIP14 is expressed on the basolateral blood-facing side of these cells. Also, when ZIP14 expression was inhibited by siRNA, Mn uptake was significantly reduced. These findings suggest that ZIP14 plays a role in Mn transport from the blood to the CSF [7].

As an exporter necessary for Mn clearance from the blood, ZnT10 may be particularly important in brain Mn homeostasis at brain barriers [14,19,20]. A recent study using pan-neuronal/glial, endoderm-specific, or whole-body *Znt10* KO mouse models showed that ZnT10 expression within the brain tissue has a protective effect against Mn overload [21]. The endoderm-specific KO group, with normal ZnT10 expression in the brain, had a less elevated brain tissue Mn level than the whole-body *Znt10* KO group, leading the authors to conclude that there is a specific role for ZnT10 in protecting the brain from high Mn under overload conditions. While ZnT10 is known to be expressed in neurons and astrocytes, its presence at the brain barriers remains unexplored. This represents a significant knowledge gap, as ZnT10 may play a crucial role in controlling the efflux of Mn from the CSF or brain parenchyma into the circulating blood.

In this study, we aimed to explore the distinct roles of the blood–brain barrier and blood–CSF barrier in maintaining Mn homeostasis by examining the protein expression of ZIP8, ZIP14, and ZnT10 in isolated mouse tissues. Additionally, we used whole-body *Znt10* KO mice as a model of Mn hyperaccumulation to expand upon an in vitro study reporting ZIP14’s ability to downregulate expression when exposed to high levels of Mn [22]. We found that, in models of Mn overload caused by *Zip14* or *Znt10* KO, the CSF accumulated significantly more Mn than wild-type controls. In the case of *Zip14* KO mice, Mn accumulated in the CSF, suggesting a loss of Mn clearance from the brain due to a lack of ZIP14 in the choroid plexus. In *Znt10* KO mice, ZIP14 protein expression was measured and found to be unchanged in the choroid plexus. Although there are no known studies on ZIP14 degradation in choroid plexus epithelial cells in culture, our result in vivo is contrary to previous work in hepatocytes in culture [22]. This also demonstrates that choroid plexus ZIP14 may not be able to control Mn homeostasis in the CSF, particularly when blood Mn rises to supraphysiological levels.

## 2. Results

### 2.1. Isolation and Validation of Choroid Plexus and Microvascular Tissues for Mn Transporter Analysis in the Brain

**Figure 1:**
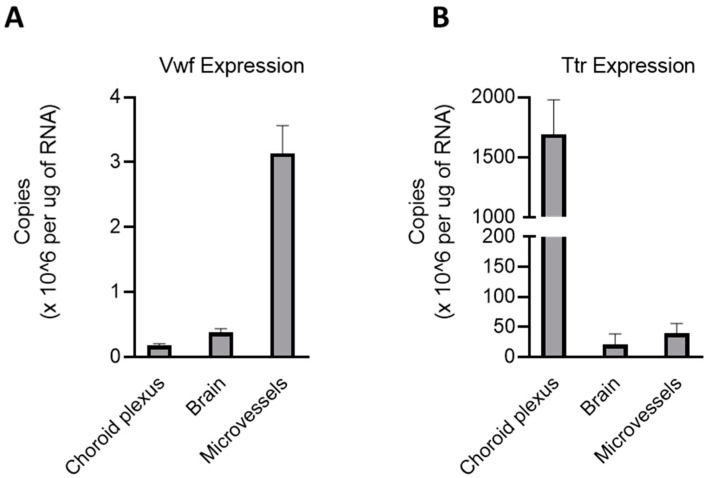
Isolation of brain barrier tissues. (**A**) Microvessels were isolated from mouse cerebral cortex and were found to be enriched in *von Willebrand Factor* (*Vwf*). This confirms that the isolated cells were primarily from the brain endothelium. (**B**) Choroid plexus tissues were isolated from the lateral ventricles and fourth ventricle. Tissues were highly enriched in *Transthyretin* (*Ttr*), a marker specific to the choroid plexus epithelium in the brain. *n* = 10 for each tissue [5 males (M) and 5 females (F)].

The choroid plexus and microvascular tissue in the brain make up two barriers that separate systemic blood circulation from the brain parenchyma. Previous research points strongly to ZIP8, ZIP14, and ZnT10 as the Mn transporters most responsible for Mn homeostasis. However, to our knowledge, no previous studies have systematically examined the protein levels of ZIP8, ZIP14, and ZnT10 in these crucial barriers. In order to understand the transport of Mn and other metals into and out of the brain, we sought to identify the relevant Mn transporters in both tissues. Microvessels were isolated from both hemispheres of the cerebral cortex of 6–8-week-old wild-type mice. Choroid plexus tissue was isolated from the fourth and both lateral ventricles. To confirm the successful isolation of these specific tissues, we used von Willebrand factor (*Vwf*) (Figure 1A) and transthyretin (*Ttr*) (Figure 1B) genes as positive controls. Within the brain, TTR is a protein exclusively secreted by choroid plexus tissue, while VWF is found in brain endothelial cells. Our results indicate that each tissue expressed a high amount of its respective marker, confirming the successful isolation of the target tissues.

### 2.2. Differential Expression of Mn Transporter Genes in the Choroid Plexus Versus Brain Microvessels

**Figure 2:**
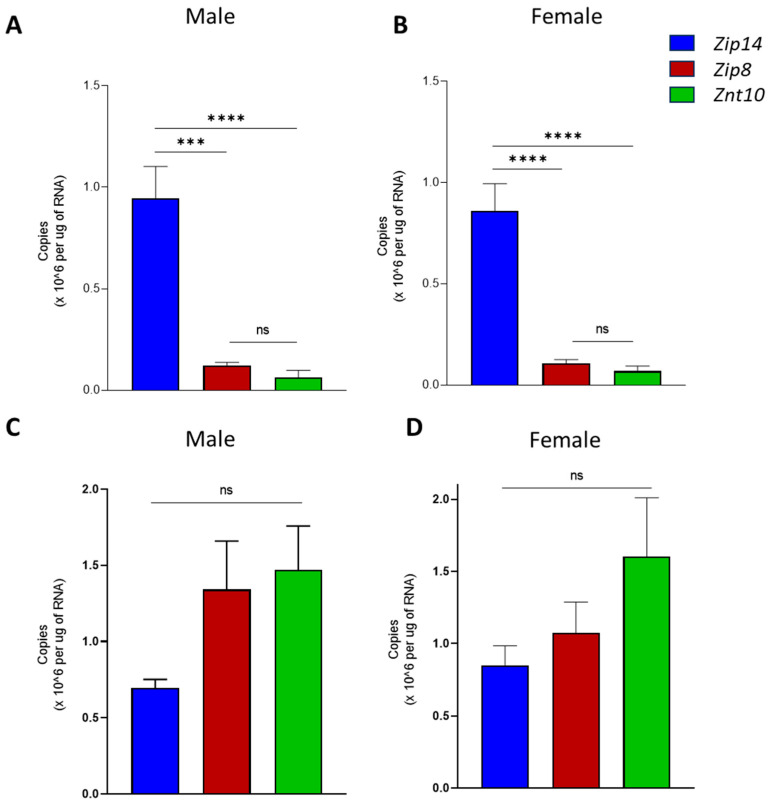
Comparison of Mn transporters’ gene expression in choroid plexus and brain microvessels. The mRNA copy numbers of *Zip8*, *Zip14*, and *ZnT10* were calculated for the choroid plexus tissues in both (**A**) male and (**B**) female mice. *Zip14* was highly expressed in the choroid plexus tissues. Both *Zip8* and *Znt10* were expressed at very low levels in the choroid plexus. Gene expression was quantified in microvessel tissue from (**C**) male and (**D**) female mice. Male microvascular tissue expressed a similar amount of *Zip8* and *Zip14* as *Znt10*. Females also had similar levels of all three transporter genes in the isolated microvessels. *n* = 10 microvessels and 10 choroid plexus tissues (5M, 5F). One-way ANOVA with Tukey’s multiple comparisons test was used to identify significant differences in expression between each gene within the tissue. (*** *p* < 0.001, **** *p* < 0.0001, ns = not significant).

Next, RNA extracted from the isolated tissues was used in qPCR analysis to compare the expression levels of each Mn transporter gene between the choroid plexus and brain microvessels. As these tissues represent two key brain barriers involved in nutrient uptake and excretion, the expression profile will help to reveal which Mn transporters play a role in Mn homeostasis at these barriers. We found that choroid plexus tissue expresses *Zip14* but has minimal *Znt10* or *Zip8* expression (Figure 2A,B). In the brain microvessels, however, *Zip14* gene copies were similar to the choroid plexus, and *Znt10* and *Zip8* expression appeared higher in males but were not significantly different (*p* = 0.11 and *p* = 0.20, respectively) (Figure 2C). In female mice, there were also no significant differences in gene expression in the microvessel tissues (Figure 2D).

**Figure 3:**
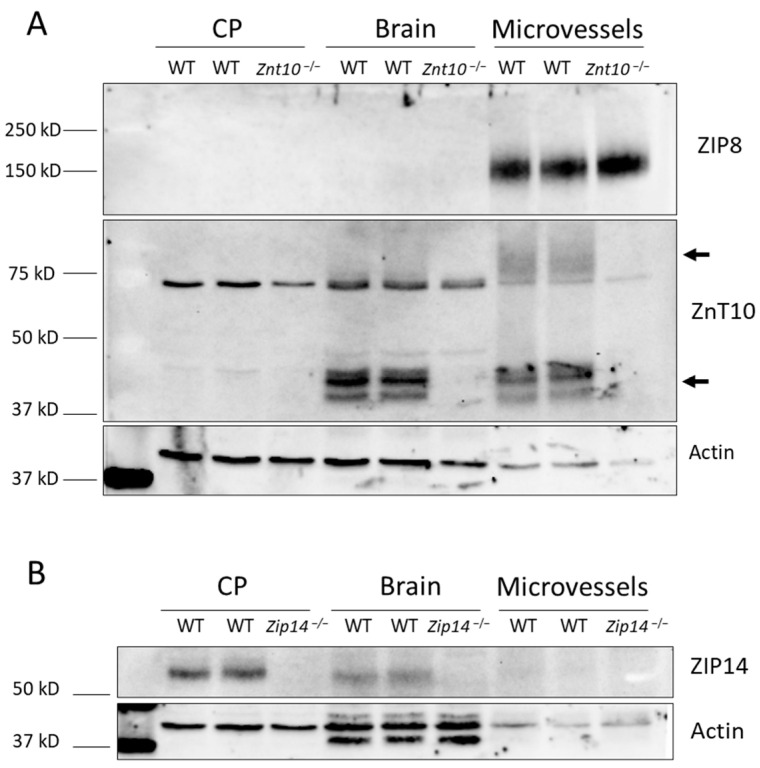
ZIP8 and ZNT10 are expressed in brain microvessels, while ZIP14 is enriched in the choroid plexus. (**A**) Immunoblotting analysis with wild-type (WT) and *Znt10* knockout (*Znt10*^−/−^) tissues indicates that the ZIP8 transporter is highly expressed in the microvascular tissue of the brain but cannot be detected in the whole brain tissue or choroid plexus (CP). Similarly, ZnT10 is expressed in the brain microvessels but not in the choroid plexus. ZnT10 is also highly expressed in the whole brain lysate. Two arrows were used to indicate the expected band pattern of the ZnT10 transporter. We used tissues from *Znt10*^−/−^ mice to show the specificity of the anti-ZnT10 antibody. (**B**) An analysis of wild-type and ZIP14 knockout (*Zip14*^−/−^) tissues indicates that ZIP14 is not detected in the microvascular tissue but is enriched in the choroid plexus. We used tissues from *Zip14*^−/−^ mice to show the specificity of the anti-ZIP14 antibody. Actin was used as the loading control. We loaded equal amounts of protein for each lane. Residual staining from a previous antibody appears as nonspecific bands above and below the Actin antibody in brain tissue wells only in panel (**B**). Actin expression is lower in brain microvessels compared to the whole brain and CP.

To further investigate the role of these transporters, we assessed protein levels by immunoblotting on both brain microvessel and choroid plexus tissues. The calculated molecular weights of ZIP8 and ZnT10 were both around 50 kD, but validated antibodies from our lab showed ZIP8 at around 150 kD, ZnT10 at 37–50 kD, and a smeared band between 75 and 100 kD. The calculated molecular weight of ZIP14 was 55 kD, consistent with the smeared band we consistently observed in our experiments. Our analysis revealed that ZnT10 and ZIP8 were present in the microvessels but were not detectable in the choroid plexus (Figure 3A). Conversely, the ZIP14 protein was found to be enriched in the choroid plexus but was not detectable in the brain microvessels (Figure 3B). These findings suggest that distinct Mn transporters are involved in different brain barriers: ZIP8 and ZnT10 are implicated in Mn import and export in the brain microvessels, respectively, while ZIP14 may play a role in Mn transport within the choroid plexus.

### 2.3. ZIP14 Localizes to the Basolateral Membrane of Choroid Plexus Epithelium

**Figure 4:**
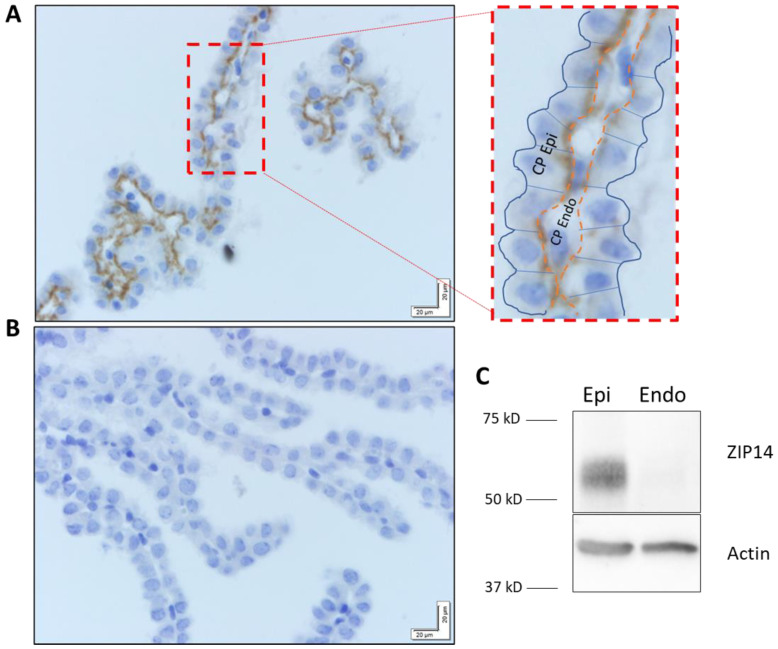
ZIP14 localizes to the basolateral membrane of choroid plexus epithelial cells. (**A**) Immunohistochemical staining results in antibody-specific signal from ZIP14 along the basolateral side [orange dashed line] of the continuous choroid plexus epithelial layer [CP Epi]. (**B**) In *Zip14*^−/−^ mouse CP tissue, no signal is observed. (**C**) Cultured primary cells from the epithelial (Epi) and endothelial (Endo) layers of human choroid plexus tissue were used for ZIP14 detection by immunoblotting. ZIP14 was only present in epithelial cells. Actin was used as the loading control.

The tight junction-connected epithelial cells of the choroid plexus form the blood–CSF barrier. However, the whole choroid plexus tissues collected also contain endothelial cells. Immunohistochemical staining of ZIP14 in the choroid plexus shows the apparent expression of ZIP14 along the basolateral side of the choroid plexus epithelial cells (Figure 4A). The specificity of the ZIP14 antibody was confirmed with staining of tissue from *Zip14* KO mice (Figure 4B). Given the close proximity of the endothelial and epithelial layers, we aimed to confirm that ZIP14 expression is restricted to epithelial, rather than endothelial, expression. To achieve this, we used two primary cell lines derived from the human choroid plexus: an epithelial (HCPepiC) and an endothelial (HCPEC) population. Both cell lines were probed for ZIP14, and it was confirmed that ZIP14 was not detectable in the choroid plexus endothelial cell line (HCPEC) (Figure 4C). In addition to the in vitro evidence supporting epithelial-specific ZIP14 expression, the staining pattern for ZIP14 in choroid plexus tissue is consistent with the known morphology of the choroid plexus epithelium—continuous, polarized, and uniform (Figure 4A). In contrast, the blood vessels of the choroid plexus endothelium vary in size and spacing and are not likely to form a continuous layer in tissue sections. Together, the results from both primary cell data and the choroid plexus immunostaining indicate that ZIP14 is expressed along the continuous basolateral side of the choroid plexus epithelium. The localization of ZIP14 at the choroid plexus epithelium suggests its involvement in regulating metal transport across the blood–CSF barrier, thereby playing a role in maintaining Mn levels in the CSF.

### 2.4. ZIP14 Expression in the Choroid Plexus Is Unchanged in a Mouse Model of Mn Overload

**Figure 5:**
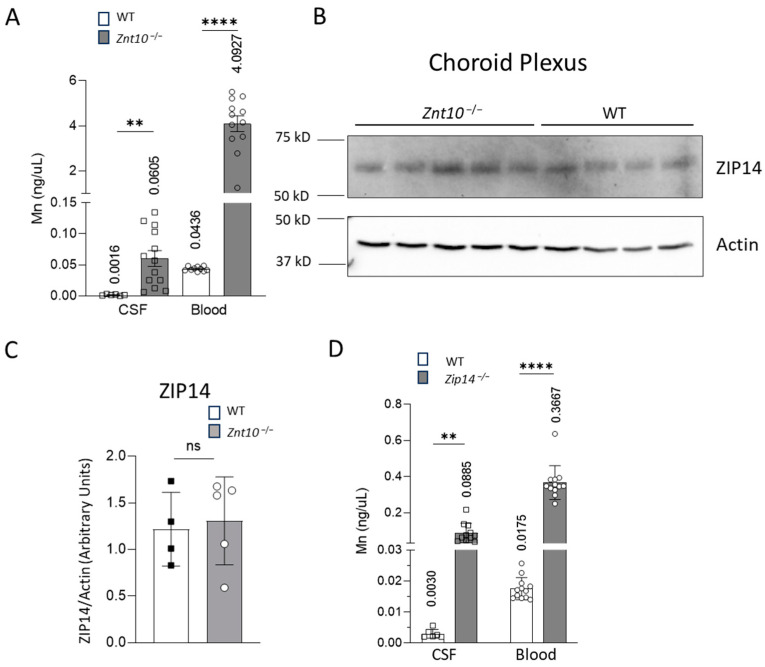
ZIP14 expression in the choroid plexus does not change in Znt10^−/−^ mice. (**A**) *Znt10*^−/−^ mice accumulate Mn in the CSF at levels more than 30 times higher than WT mice (Blood *n* = 5–6 male, 5–6 female; CSF n = 3–6 male, 3–6 female). (**B**) Despite a large increase in CSF and blood Mn in the *Znt10*^−/−^ mice, the expression levels of ZIP14 do not change (*n* = 3F, 2M KO; *n* = 4M WT). (**C**) Quantification of ZIP14 band shown in panel (**B**). (**D**) In *Zip14*^−/−^ mice, CSF accumulated Mn at a similarly high level to that of *Znt10*^−/−^ mice (Blood *n* = 6 male, 6 female; CSF *n* = 3–5 male, 3–6 female). The mean values for each group are shown above the corresponding bar. Individual *t*-tests were conducted to compare the means of WT and KO values for each body fluid (** *p* < 0.01, **** *p* < 0.0001, ns = not significant).

Given that ZIP14 is crucial for Mn homeostasis and is localized to the choroid plexus epithelial layer, examining its regulation can provide insights into how disturbance in Mn levels may affect the function of ZIP14. To address this, we used a Mn overload mouse model, *Znt10* KO mouse, to assess the impact of high Mn on ZIP14 expression. We first measured the Mn levels in CSF and blood from wild-type and *Znt10* KO mice. While it has been established that ZnT10 deficiency leads to increased Mn in the blood and whole brain [21,23,24], its effect on Mn levels in the CSF remains unknown. By measuring Mn concentrations in the CSF and blood, we aimed to understand Mn distribution at the basolateral or apical side of the blood–CSF barrier. We found that *Znt10* KO mice accumulated more than 30 times more Mn in the CSF than the wild-type littermates (Figure 5A). Furthermore, immunoblotting results showed that the expression of ZIP14 in the choroid plexus is unchanged in *Znt10* KO mice (Figure 5B,C). Since ZIP14 is highly expressed at the blood–CSF barrier in wild-type and *Znt10* KO mice, but is not present in *Zip14* KO mice, we then aimed to determine whether the lack of ZIP14 at the blood–CSF barrier leads to a higher accumulation of Mn in the CSF. To do this, we collected CSF from *Zip14* KO mice and their littermate controls. Interestingly, the *Zip14* KO mice accumulate a similar level of Mn in the CSF (Figure 5D).

## 3. Discussion

Previous studies indicate the importance of Mn transporters ZIP8, ZIP14, and ZnT10 in systemic Mn homeostasis in humans [25,26,27,28,29] and mice [30,31,32]. Studies with gene KO mouse models further elucidate the mechanism of Mn homeostasis within specific organs [30,31,32]. However, there remains a critical gap in our understanding of the specific roles these proteins play within the brain. The function of these transporters is closely tied to their precise localization within brain tissues, making it essential to investigate their distribution and expression to fully grasp their impact on brain Mn homeostasis. We found that all three transporters are expressed on either the microvessels (blood–brain barrier) or choroid plexus (blood–CSF barrier), with no apparent overlap in protein expression between the two tissues.

Gene expression analysis shows that brain microvessels are enriched in *Zip8* and *Znt10* mRNA, with *Zip14* mRNA being moderately less abundant. In contrast, choroid plexus tissues express significantly more *Zip14* mRNA compared to *Zip8* or *Znt10*, with the latter two being approximately 10% of *Zip14* mRNA levels. The purity of the tissue samples representing each brain barrier was confirmed by measuring the copy number of tissue-specific genes *Ttr* and *Vwf*, ensuring that the choroid plexus and microvessel tissues were cleanly isolated and strongly expressed their respective tissue-specific proteins. These data are consistent with the previous gene expression data of *Zip8* in the brain blood vessels [8]. In addition, our present study provides a comparison of copy numbers for each gene across both brain barriers, allowing us to identify which transporter is more abundant in each tissue.

Protein expression of each transporter, as detected by immunoblotting, was somewhat consistent with the mRNA expression data. ZnT10 and ZIP8 were expressed in microvascular tissue but absent from the choroid plexus. On the other hand, ZIP14 was found in the choroid plexus tissue but was undetectable in brain microvessels, suggesting the involvement of ZIP14 in Mn metabolism across the choroid plexus epithelium. As a basolateral Mn transporter in the intestinal and hepatic epithelial cells, ZIP14 is believed to transport Mn unidirectionally from the blood circulation to epithelium of the tissue [31]. Our study confirmed that ZIP14 is exclusively present along the basolateral membrane of the choroid plexus epithelium. Interestingly, ZIP14 expression remains unchanged under high brain Mn conditions, as it was comparable in *Znt10* KO mice and their wild-type counterparts.

Manganese accumulation in the brain has been a heavily-reported consequence of high blood Mn levels. In the two mouse models of Mn overload, *Zip14* KO and *Znt10* KO, previous studies have reported whole-brain and dissected-brain Mn levels to indicate the severity of Mn accumulation. Here, we report that *Zip14* KO mice have similar levels of Mn loading in the CSF as *Znt10* KO mice, when compared to their respective wild-type littermate group. This finding indicates that ZIP14 is not necessary for Mn accumulation in the CSF under high Mn conditions, as other transport mechanisms may compensate for its absence.

There were some limitations in this study, primarily stemming from the use of immunoblotting to detect proteins. We chose to use the ZIP8, ZIP14, and ZnT10 antibodies that were made by our lab because they have been validated using immunoblotting and knockout tissue samples. Multi-pass transmembrane proteins, such as the ZIP and ZnT proteins, can have isoforms and subunits that appear as smears or a series of bands in an immunoblot. We are confident in the validity of these antibodies because the band pattern has been compared to mouse tissues with the protein of interest knocked out. In Figure 3A, ZnT10 is seen as a faint, smeared band around 75–100 kD and a series of smaller bands between 37 and 50 kD, despite the reported molecular weight of 50 kD (Uniprot ID: Q3UVU3). Another limitation of immunoblotting is a lack of sensitivity when using antibodies to detect a single protein in a tissue lysate. In our study, we could strongly detect the presence of ZIP8 protein in isolated blood vessels, but we could not detect ZIP8 in whole-brain lysate. We attribute this to the fact that only a small fraction of the total brain lysate is made up of endothelial cells, and ZIP8 may not be expressed at a detectable level in neurons and glia, so the total number of cells expressing ZIP8 is minimal. Because immunoblotting cannot explicitly determine whether a protein is expressed or not in a tissue lysate, we are careful to describe the presence of each Mn transporter as “detectable”, “enriched”, or “not detected” in each sample. We cannot say with any certainty that ZIP14 is not expressed in brain microvessels, or ZIP8 and ZnT10 are not expressed in the choroid plexus.

In summary, by identifying the distinct localization of ZIP8, ZIP14, and ZnT10 in the blood–brain barrier and blood–CSF barrier, our study highlights the specialized roles these transporters play in maintaining Mn homeostasis in different brain regions. The observation that ZIP14 is exclusively present in the choroid plexus, but not detected in the brain microvessels, suggests a potential role for ZIP14 in Mn transport across the blood–CSF barrier. However, our additional findings indicate that even in the absence of ZIP14, Mn can still enter the CSF when systemic levels are elevated. This suggests that while ZIP14 may facilitate Mn transport under normal conditions, other transport mechanisms or compensatory pathways are likely activated when Mn levels are increased, ensuring Mn homeostasis in the CSF. In the future we intend to investigate the potential changes in ZIP8 or ZnT10 expression in another model of Mn hyperaccumulation. This insight will help to elucidate how Mn transport is regulated in the brain, and it underscores the potential for compensatory mechanisms involving other transporters in cases of Mn overload, which could inform future research and therapeutic strategies for managing Mn-related disorders.

## 4. Materials and Methods

### 4.1. Isolation of Brain Barrier Tissues

All mice were housed at the University of Arizona’s central animal facility, and all procedures were approved by the Institutional Animal Care and Use Committee. Mice were maintained on a 12 h light/dark schedule and received water and standard rodent diet (Teklad 7913, Envigo, Indianapolis, IN, USA).

Animal tissues were collected from male and female mice at 6–9 weeks old. Mice were anesthetized with Ketamine/Xylazine and exsanguinated via cardiac puncture. For the isolation of the choroid plexus tissues, the whole brain was removed and placed in chilled phosphate-buffered saline. Under a dissecting microscope, meninges were removed, and watchmakers’ forceps were used to partially separate the brain hemispheres at the longitudinal fissure. The cortex was lifted from the hippocampus to expose each lateral ventricle with a piece of choroid plexus inside. The tissues were collected by gently pulling each choroid plexus segment out of the ventricle using the watchmakers’ forceps. The fourth ventricle choroid plexus was then isolated by lifting the cerebellum from the brain stem and exposing the tissue. The isolated tissues were either stored in DNA/RNA Shield (Zymo Research, Irvine, CA, USA) at −80 °C until RNA isolation or snap-frozen in liquid nitrogen for protein analysis. Brain microvessels were isolated using a modified version of a protocol by Lee et al. [33]. Briefly, the cortex of each mouse was homogenized in MCDB 131 media and centrifuged to remove debris; then, the pellet was resuspended in PBS with 15% dextran-70. After centrifugation, the microvessel-enriched pellet was separated from the neuron- and glia-rich supernatant. The microvessel pellets were either stored in a DNA/RNA shield at −80 °C until RNA was isolated or stored at −80 °C until protein analysis was completed.

Cerebrospinal fluid was collected after exsanguination, using an established protocol [34]. Briefly, the skin and muscle were cut to expose the base of the skull and the cisterna magna. A fine-point glass capillary syringe was used to puncture the dura and extract about 10 uL of CSF from each animal. Samples were collected under a microscope and were considered free of blood if no blood vessels were damaged during fluid collection. The final CSF samples were clear and colorless. Samples were then snap-frozen in liquid nitrogen and stored at −80 °C for further use.

### 4.2. RNA Isolation and Quantitative PCR (qPCR)

qPCR data were reported as an estimated transcript copy number, similar to a method used previously [35]. A Quick-RNA Miniprep plus kit (Zymo Research, Irvine, CA, USA) was used to isolate RNA from each tissue sample. RNA concentration was measured using Nanodrop 2000 (Thermo Fisher Scientific, Waltham, MA, USA). cDNA was synthesized with the M-MuLV Reverse Transcriptase kit (New England BioLabs, Ipswich, MA, USA). qPCR was performed using 2× SYBR Green Master Mix (Thermo Fisher Scientific, Waltham, MA, USA) and primers for *Zip14* (Forward: 5′ CTCTGGAGACCTCTTTGCGG 3′; Reverse: 5′ AGAATGGTGGGGCAGAACTC 3′), *Zip8* (Forward: 5′ GGCTGACATAGACAGTAGAGGC 3′; Reverse: 5′ ACCTTCGGGGCATTGAAGAG 3′), *Znt10* (Forward: 5′ GGTTGTGGTCATCACGGCTA 3′; Reverse: 5′ CTGCCAGTTACACGGGTCTT 3′), *Ttr* (Forward: 5′ AGACACTTGGCATTTCCCCG 3′; Reverse: 5′ TCAATTCTGGGGGTTGCTGA 3′), and *Vwf* (Forward: 5′ AGATGCATGGTCTTTGCGGA 3′; Reverse: 5′ CACCGTCCATTCCTGGACAA 3′). All primers were purchased from Integrated DNA technologies (Coralville, IA, USA), and qPCR was completed using Applied Biosystems Quantstudio 5 instrument and Design and Analysis Software (version 1.4) (Thermo Fisher Scientific, Waltham, MA, USA). Standards for each reaction were prepared from the primer products. Wild-type microvessel RNA was used to amplify *Zip8* and *ZnT10* primer products, while wild-type choroid plexus RNA was used to amplify the *Zip14* primer product. Briefly, PCR was completed with 2× M-PCR Opti Mix (Bimake, Houston, TX, USA). The PCR product was loaded into a 1.5% agarose gel, and the single band of DNA was excised while DNA was isolated using the Wizard SV gel and PCR clean-up system (Promega, Madison, WI, USA). DNA concentration was quantified using Nanodrop. Copies of each gene were calculated using the known number of base pairs in the amplified product. Amplified DNA products, with calculated copy numbers, were diluted into 5–8 standards to be used in the qPCR reaction. Copy numbers of each gene in samples were estimated based on the serially diluted standards that formed the standard curve.

### 4.3. Immunoblotting

Immunoblotting analysis was completed as previously described [31]. The choroid plexus tissues were lysed using a 2 mL dounce homogenizer and NETT lysis buffer (150 mM NaCl, 5 mM EDTA, 10 mM Tris, 1% TritonX-100, 1× Protease inhibitors [Bimake, Houston, TX, USA]). Isolated microvessel pellets were sonicated briefly in lysis buffer and then vortexed repeatedly on ice. Both tissues were centrifuged at 10,000× *g* to separate cell nuclei and larger debris. Protein quantification of clarified supernatant was determined using the RC DC protein assay kit (Bio-rad, Hercules, CA, USA). Equal amounts of protein samples were prepared with a 1× Laemmli buffer, heated at 37 °C for 30 min, and then loaded into wells of 7.5–10% polyacrylamide SDS gels. Proteins were electrophoretically separated at 120 V for 60–90 min. Proteins were transferred to nitrocellulose membranes for 2 h at 100 V. All blots were blocked in blocking buffer consisting of 5% nonfat milk in TBS with 0.1% Tween-20 (TBST) for one hour and then probed with anti-mZIP8 (1:1000), mZIP14 (1:1000), mZnT10 (1:1000), or hZIP14 (1:1000) primary antibodies, followed by anti-rabbit-HRP secondary antibody (Millipore Sigma, Burlington, MA, USA) (1:4000). Horseradish peroxidase (HRP)-conjugated Beta-Actin antibody (Proteintech, Rosemont, IL, USA) (1:20,000) was used to probe for normalization proteins. All primary antibodies were made in house using the same process as our published protocol for the ZIP8 antibody generation [36]. Blots were imaged with the ChemiDoc MP imaging system and Image Lab software version 5.2.1 (Bio-Rad, Hercules, CA, USA).

### 4.4. Immunohistochemistry

Slides of paraffin-embedded choroid plexus tissues were prepared by UAC Pathology Services at the University of Arizona. 3,3′-Diaminobenzidine (DAB) staining was completed by the Tissue Acquisition and Cellular/Molecular Analysis Shared Resource at the University of Arizona. Briefly, tissues were heated and dewaxed at 72 °C, and heat-induced epitope retrieval was performed. The rest of the blocking steps were performed at room temperature. After blocking with peroxide for 5 min, slides were washed and then incubated with a 1:75 dilution of mZIP14 antibody for 15 min, followed by an HRP-conjugated rabbit secondary antibody incubation for 8 min. Then, slides were visualized with DAB for 10 min and counterstained with hematoxylin.

### 4.5. Cell Culture

Primary human choroid plexus epithelial cells (HCPEpiC, Cat# 1310) and primary human choroid plexus endothelial cells (HCPEC, Cat# 1300) were purchased from ScienCell (Carlsbad, CA, USA). Both cell lines were thawed and grown in DMEM/F12 50:50, supplemented with epithelial or endothelial growth supplements (Sciencell), 10% FBS, and 1× Penicillin/Streptomycin. Cells were cultured for 96 h before they were pelleted and lysed in Na-EDTA Tris + 1% Triton-x lysis buffer with protease inhibitors.

### 4.6. Metal Level Measurement by Inductively Coupled Plasma Mass Spectrometry (ICP-MS)

Digestion of all samples was performed in concentrated nitric acid for 16 h at 80 °C. Samples were diluted in Milli-Q water to a final concentration of 3% nitric acid and analyzed by ICP-MS at the Arizona Laboratory for Emerging Contaminants (ALEC). The volumes for each biological fluid are described as follows: samples containing 50 µL of blood were used for each individual mouse. Then, 10 µL of CSF was collected from each mouse, and individual samples were analyzed for all *Znt10* KO and *Zip14* KO mice. For wild-type controls, Mn levels of individuals were below the detectable limit of the mass spectrometer, so three groups of pooled samples totaling 80 µL of CSF were analyzed as the control.

## Data Availability

We do not have any additional data beyond the manuscript and the Supplemental Figures. This part is not applicable to our paper.

## Supplementary Materials

The following supporting information can be downloaded at: https://www.mdpi.com/article/10.3390/ijms251910342/s1.
