# Supplementary material for: Expression of Manganese Transporters ZIP8, ZIP14, and ZnT10 in Brain Barrier Tissues
Source: Int J Mol Sci. 2024 Sep 26;25(19):10342. doi: 10.3390/ijms251910342 (PMC11476488; doi:10.3390/ijms251910342)

Supplemental Figures

Expression of Manganese Transporters ZIP8, ZIP14, and ZnT10 in Brain Barrier Tissues

Shannon Morgan McCabe [1] and Ningning Zhao [1*]

[1] School of Nutritional Sciences and Wellness, The University of Arizona Tucson, AZ 85721, USA

* Correspondence: zhaonn@arizona.edu

Figure S1. Comparison of gene expression between two tissues. Student's *t*-test was used to compare the means of microvessel gene expression to choroid plexus gene expression in both male and female mice. *p<0.05, ** p <0.01, ns=not significant.

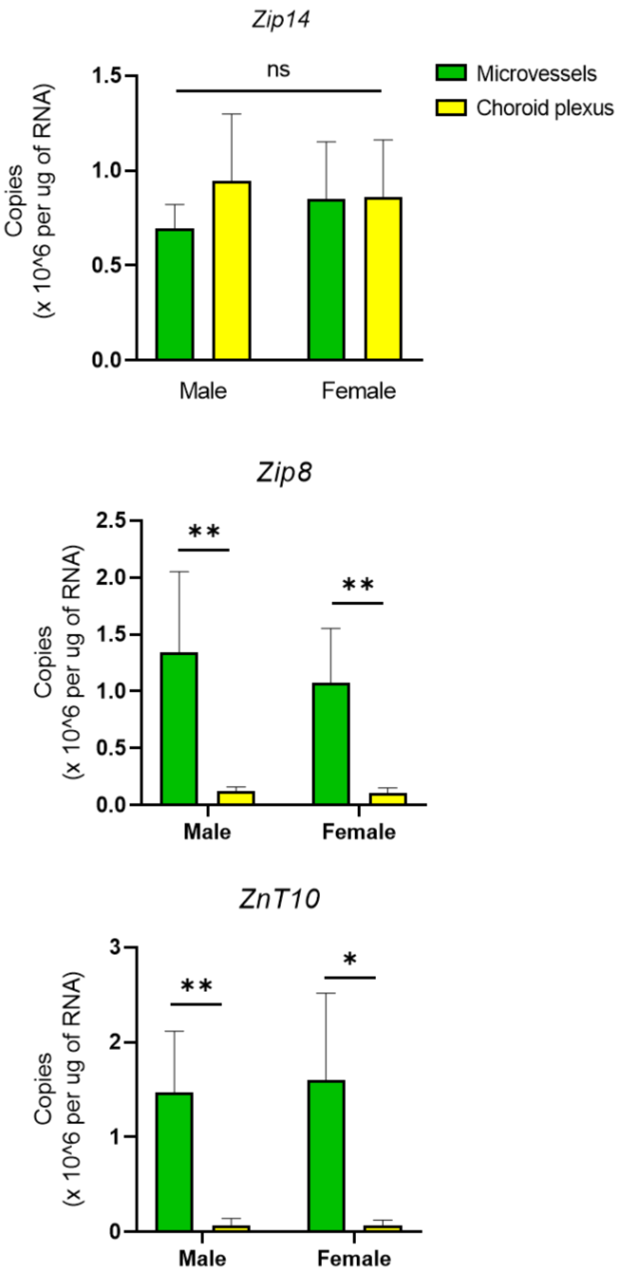

Figure S2. Uncropped Western blot images for Figure 3A

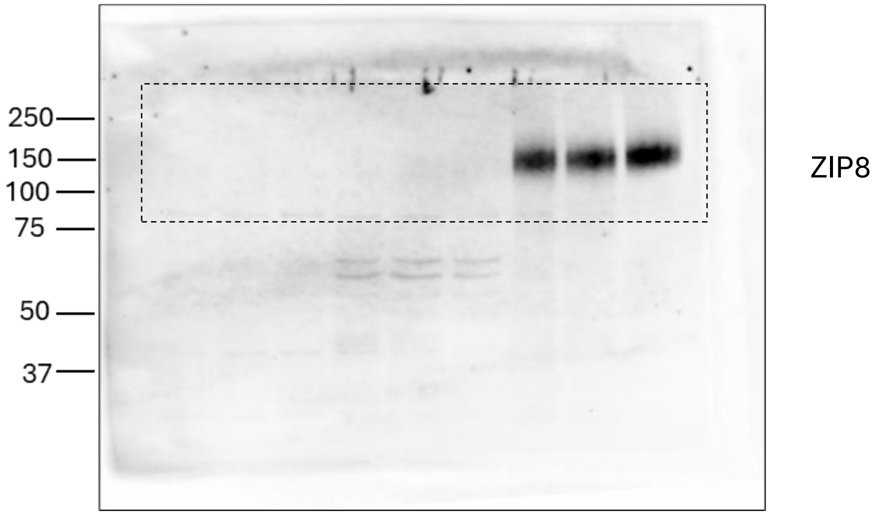

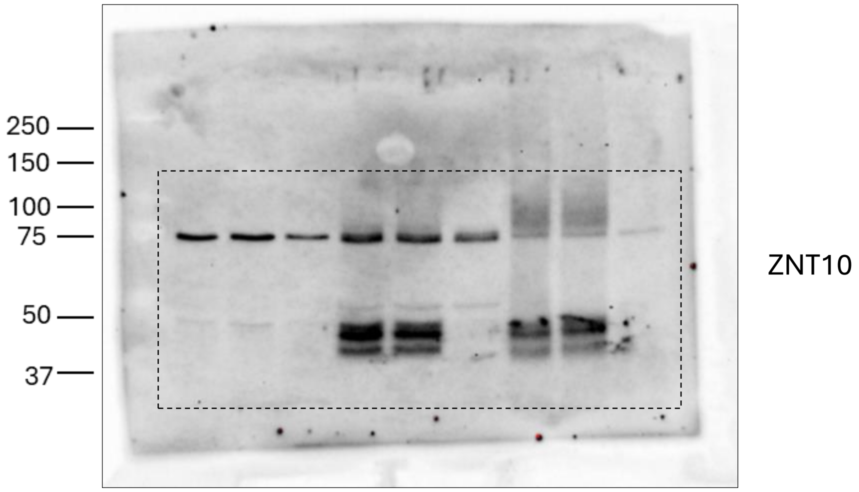

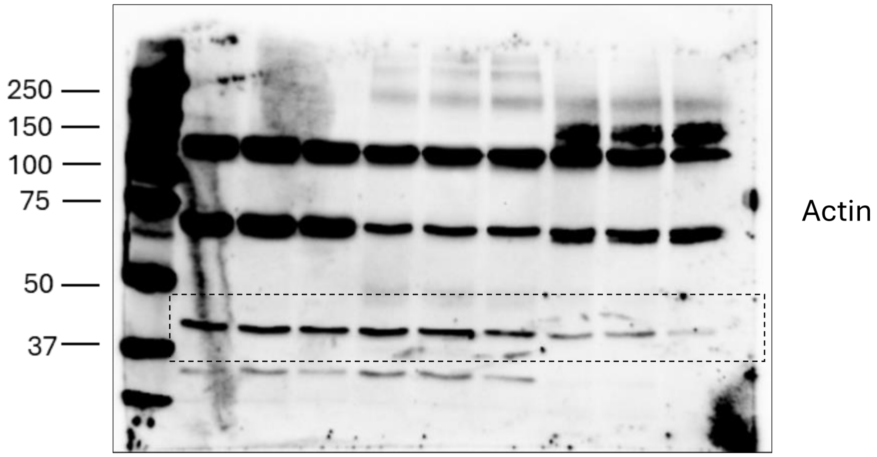

Figure S3. Uncropped Western blot images for Figure 3B

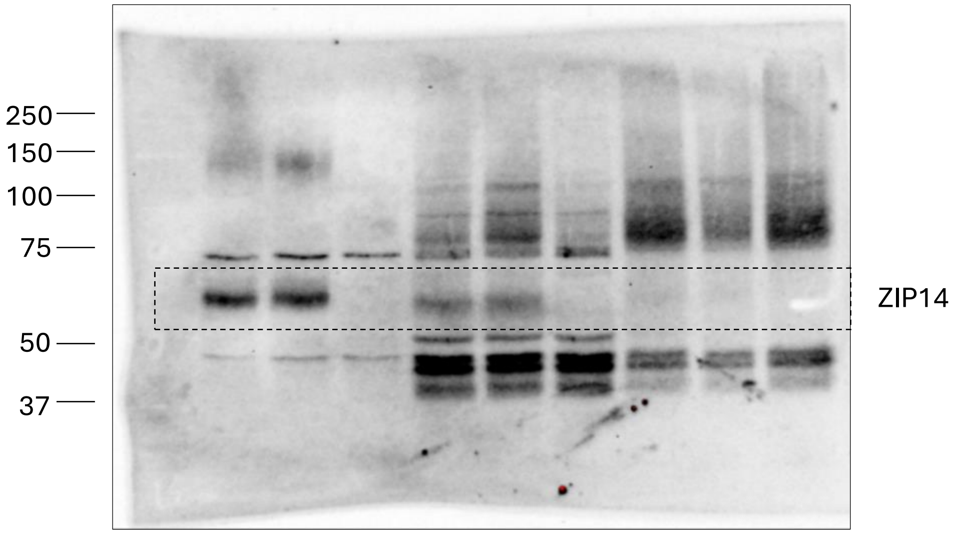

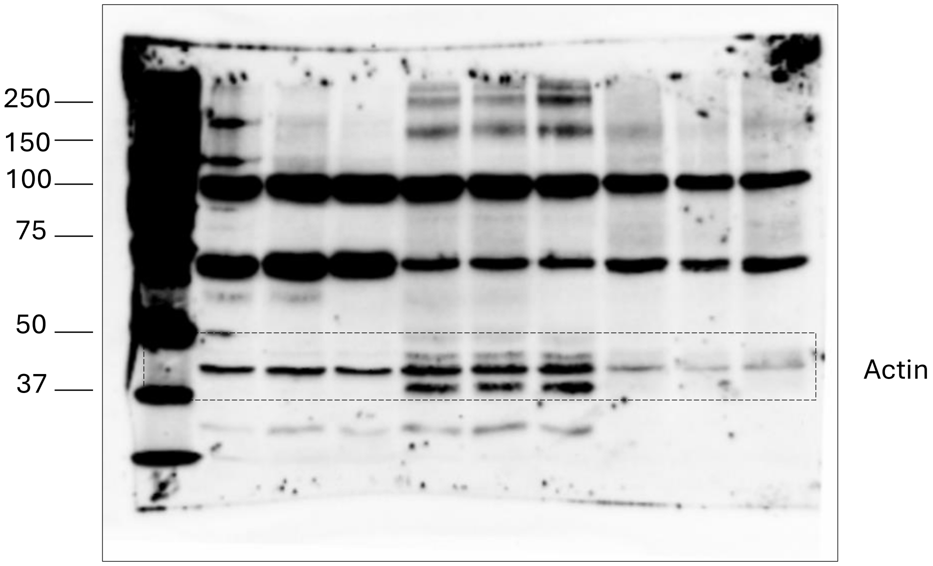

Figure S4. Uncropped Western blot images for Figure 4C and quantification of ZIP14 band shown in Figure 4C

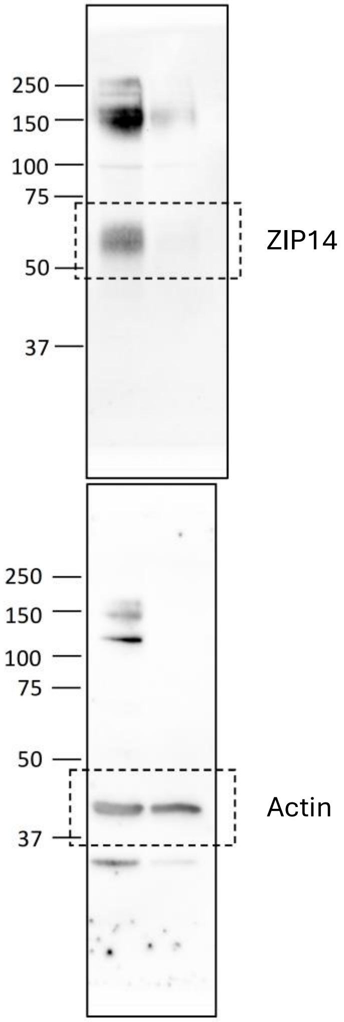

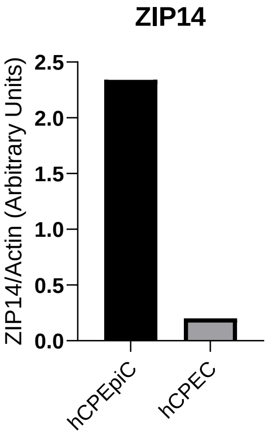

Figure S5. Uncropped Western blot images for Figure 5B

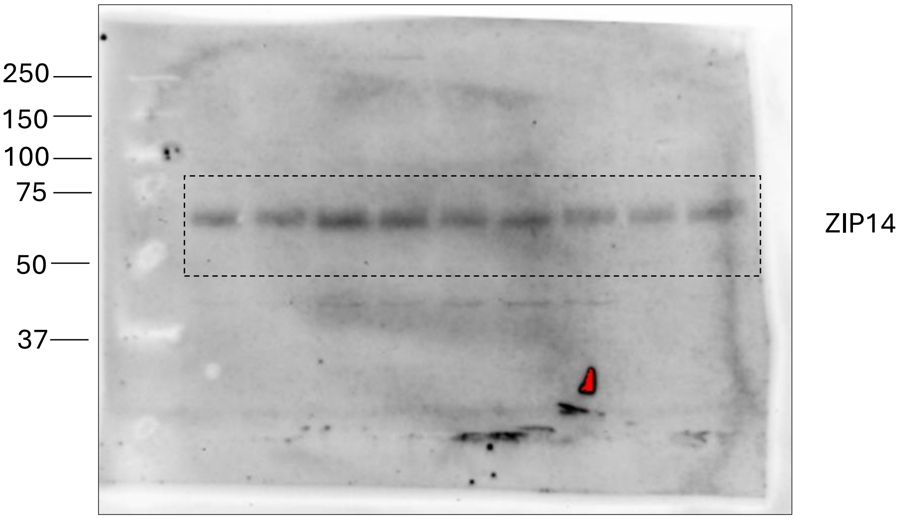

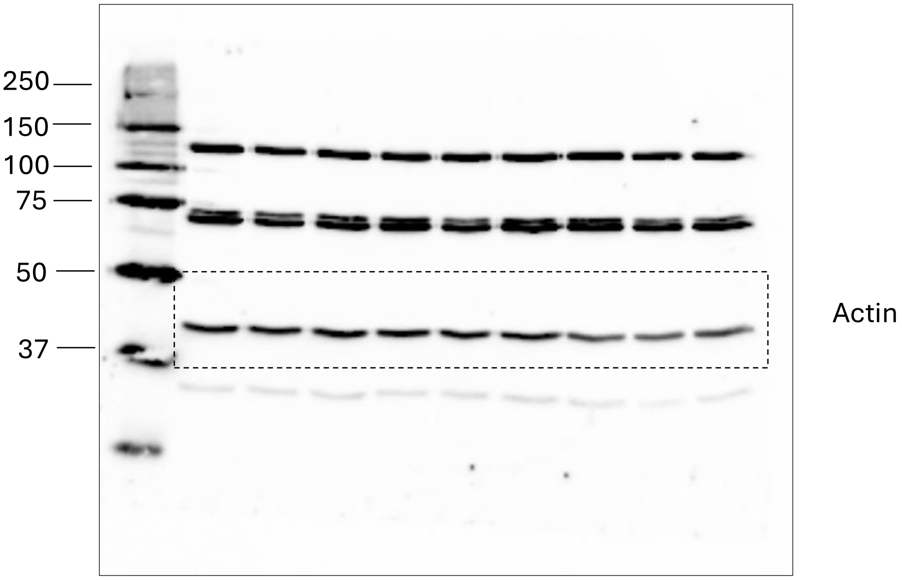

Supplement: Supplementary file 1 [file ijms-25-10342-s001.zip › ijms-3192697-supplementary.pdf]
